# Evaluating and optimizing the consolidated framework for implementation research (CFIR) for use in low- and middle-income countries: a systematic review

**DOI:** 10.1186/s13012-020-0977-0

**Published:** 2020-03-12

**Authors:** Arianna Rubin Means, Christopher G. Kemp, Marie-Claire Gwayi-Chore, Sarah Gimbel, Caroline Soi, Kenneth Sherr, Bradley H. Wagenaar, Judith N. Wasserheit, Bryan J. Weiner

**Affiliations:** 1 Department of Global Health, University of Washington, Seattle, WA USA; 2 Department of Child, Family and Population Health Nursing, University of Washington, Seattle, WA USA; 3 Department of Industrial & Systems Engineering, University of Washington, Seattle, WA USA; 4 Department of Epidemiology, University of Washington, Seattle, WA USA; 5 Fred Hutchinson Cancer Research Center, University of Washington, Seattle, Washington USA; 6 Department of Medicine, University of Washington, Seattle, Washington USA; 7 Department of Health Services, University of Washington, Seattle, Washington USA

**Keywords:** Consolidated Framework for Implementation Research, Global health, Health systems, Systematic review

## Abstract

**Background:**

The Consolidated Framework for Implementation Research (CFIR) is a determinants framework that may require adaptation or contextualization to fit the needs of implementation scientists in low- and middle-income countries (LMICs). The purpose of this review is to characterize how the CFIR has been applied in LMIC contexts, to evaluate the utility of specific constructs to global implementation science research, and to identify opportunities to refine the CFIR to optimize utility in LMIC settings.

**Methods:**

A systematic literature review was performed to evaluate the use of the CFIR in LMICs. Citation searches were conducted in Medline, CINAHL, PsycINFO, CINAHL, SCOPUS, and Web of Science. Data abstraction included study location, study design, phase of implementation, manner of implementation (ex., data analysis), domains and constructs used, and justifications for use, among other variables. A standardized questionnaire was sent to the corresponding authors of included studies to determine which CFIR domains and constructs authors found to be compatible with use in LMICs and to solicit feedback regarding ways in which CFIR performance could be improved for use in LMICs.

**Results:**

Our database search yielded 504 articles, of which 34 met final inclusion criteria. The studies took place across 21 countries and focused on 18 different health topics. The studies primarily used qualitative study designs (68%). Over half (59%) of the studies applied the CFIR at study endline, primarily to guide data analysis or to contextualize study findings. Nineteen (59%) of the contacted authors participated in the survey. Authors unanimously identified *culture* and *engaging* as compatible with use in global implementation research. Only two constructs, *patient needs and resources* and *individual stages of change* were commonly identified as incompatible with use. Author feedback centered on team level influences on implementation, as well as systems characteristics, such as health system architecture. We propose a “Characteristics of Systems” domain and eleven novel constructs be added to the CFIR to increase its compatibility for use in LMICs.

**Conclusions:**

These additions provide global implementation science practitioners opportunities to account for systems-level determinants operating independently of the implementing organization. Newly proposed constructs require further reliability and validity assessments.

**Trial registration:**

PROSPERO, CRD42018095762

Contributions to the literature

- The Consolidated Framework for Implementation Research (CFIR) is a widely used determinants framework. It was conceived in a high-income country; however, determinants of implementation may be substantially different in low- and middle-income countries (LMICs).
- We found most CFIR constructs compatible with use in LMICs. However, the CFIR was often applied in LMICs to address research questions above the outer setting and regarding health systems functionality.
- There may be key constructs and domains that could augment the CFIR for use in LMICs. Specifically, we propose a Characteristics of Systems domain that characterizes broader health systems and geopolitical factors influencing implementation, scalability, and sustainability.

## Introduction

Implementation scientists practicing in both low- and middle-income countries (LMICs) and high income countries (HICs) increasingly use theories, models, and frameworks to optimize study design, data collection, analysis, and dissemination [1]. These guiding tools are intended to enhance the generalizability of findings by establishing common concepts and terminologies that can be applied across disparate research studies and settings. Due to its comprehensiveness and flexibility, the Consolidated Framework for Implementation Research (CFIR) is a popular framework that presents a taxonomy for conceptualizing and distinguishing between a wide spectrum of contextual determinants of implementation success, ranging from external implementation context to innate intervention characteristics [2]. Damschroder and colleagues introduced the CFIR in 2009 as a meta-theoretical framework compiling nineteen preceding implementation theories [2]. The CFIR presents five domains categorizing 39 constructs and provides a repository of standardized factors that influence implementation effectiveness [2]. The domains and constructs are intended to characterize the entirety of the implementation process (Appendix 1), and researchers are expected to select constructs that resonate with a particular research question. The CFIR is thus considered a “determinants framework” in that it can be applied with deductive reasoning to identify barriers and enablers that influence targeted implementation outcomes [1].

A 2016 systematic review identified 26 meaningful applications of the CFIR across a wide range of topic areas and acknowledged a number of opportunities to improve application of the CFIR across the research spectrum [3]. Notably, only two studies (8%) included in the systematic review took place in an LMIC (Kenya), with the remaining studies taking place in the USA, Canada, Sweden, the UK, and Australia. The CFIR, like most frameworks of implementation determinants, was conceived in an HIC [1, 4, 5]. However, implementation determinants might manifest differently in LMICs and HICs due to variations in health system structures, population-level morbidity and mortality profiles, resource availability, and cultural and socio-political norms. Implementation science theories, models, and frameworks, including the CFIR, may require adaptation or contextualization to fit the needs of implementation science practitioners in LMIC settings.

The purpose of this review is to report upon use of the CFIR in LMICs and provide recommendations on how the framework can be enhanced for optimal performance in implementation research in LMIC settings moving forward. Thus, three primary objectives of this review include the following: (1) to characterize the ways in which the CFIR has been applied in LMIC contexts, (2) to identify which CFIR constructs appear compatible, incompatible, or irrelevant with global implementation science research, and (3) to identify opportunities to refine the CFIR to optimize utility in LMIC settings.

## Methods

The systematic review protocol is registered in the International Prospective Register of Systematic Reviews (PROSPERO #CRD42018095762) and followed the Preferred Reporting Items for Systematic Reviews and Meta-Analyses (PRISMA) guidelines (Additional file 1) [6].

We searched Medline, CINAHL, PsycINFO, CINAHL, SCOPUS, and Web of Science from inception until April 5, 2019, to identify original peer-reviewed research in any language that cited the original CFIR publication by Damschroder and colleagues or mentioned CFIR in the title/abstract, and that took place within an LMIC. The classification of a country as an LMIC was determined based on the 2018 World Bank classification criteria [7]. The Covidence tool was used to remove duplicate studies and to conduct study screening [8]. Two reviewers (ARM and CK) reviewed all titles and abstracts independently, followed by independent full text review of remaining articles. Disagreements were resolved through discussion until consensus was reached.

During full text review, we excluded all studies that did not take place in an LMIC or were not published in peer-reviewed journals. We also excluded protocols, conference abstracts, editorials, and original research that cited the CFIR, but did not utilize the CFIR to guide study design, implementation, or analysis.

We abstracted data from each article using a standardized abstraction tool in Microsoft Excel to capture information relevant to: study location, study dates, health topic of focus, research objective, intervention, whether or not the intervention was part of a broader program or policy initiative, the target population, study design (qualitative, quantitative, or mixed methods), unit of analysis (patients/community members, providers at health facilities or in community-based programs, or organizations/health systems), phase of implementation (pre, during, or post-implementation), manner in which the CFIR was used (informing framework only, study design or formative evaluation, data collection, data analysis, interpret or contextualize findings, or multiple), CFIR domains and constructs of focus, rationale provided for selecting specific constructs, and any associations between key CFIR constructs and study outcomes (if investigated). These data abstraction categories purposively build off of and expand upon the review conducted by Kirk and colleagues to ensure comparability [3]. All articles were read in full and data were abstracted from studies by two reviewers (CK and MCGC), with a third reviewer independently reviewing and validating all data abstractions (ARM). Any discrepancies between reviewer interpretations or abstracted data were resolved via iterative group consultation until consensus was reached.

We designed a standardized questionnaire in REDCap, and sent the questionnaire to the corresponding author of most included studies (surveys were not sent to authors of studies published immediately prior to paper submission) [9]. Conference abstracts from the 2016, 2017, and 2018 AcademyHealth Annual Conferences on the Science of Dissemination and Implementation in Health were also reviewed to identify authors currently using the CFIR in LMICs whose publications were pending. The purpose of the questionnaire was to determine the following: (1) why the study authors chose the CFIR as a guiding framework for their research study, (2) which domains and constructs they found to be compatible, incompatible, or irrelevant to their research and why, and (3) ways in which the authors believe that the CFIR could be optimized or updated for use in LMIC contexts. Compatible constructs were those that were easily applied within the research study as the definition of the construct did not require any adaptation to fit the context in which the author was working. Incompatible constructs were not easily applied to the author’s research study, as the definition of the construct required significant adaption to fit the context in which they were working, or the specific topic of inquiry was not well described by the construct. Irrelevant constructs were those that were simply not pertinent to the research project at hand. Authors had the opportunity to provide further feedback about CFIR constructs and domains via open text boxes, and responses were reviewed to identify key patterns in newly proposed constructs or domains. Contacted authors were sent a reminder email if they did not initially respond to the online questionnaire within a 2-week period, with a final reminder sent 2 weeks later. If a corresponding author responded that a different study author should participate, instead that author was contacted as well.

Opportunities to optimize the CFIR were conceived through author insights in the published manuscripts, feedback from authors via the standardized questionnaires described above regarding specific recommendations for new constructs or domains, author feedback regarding challenges and theoretical gaps in the framework, as well as via the group discussion and consensus of the authors of this review.

## Results

### Systematic review

**Fig. 1:**
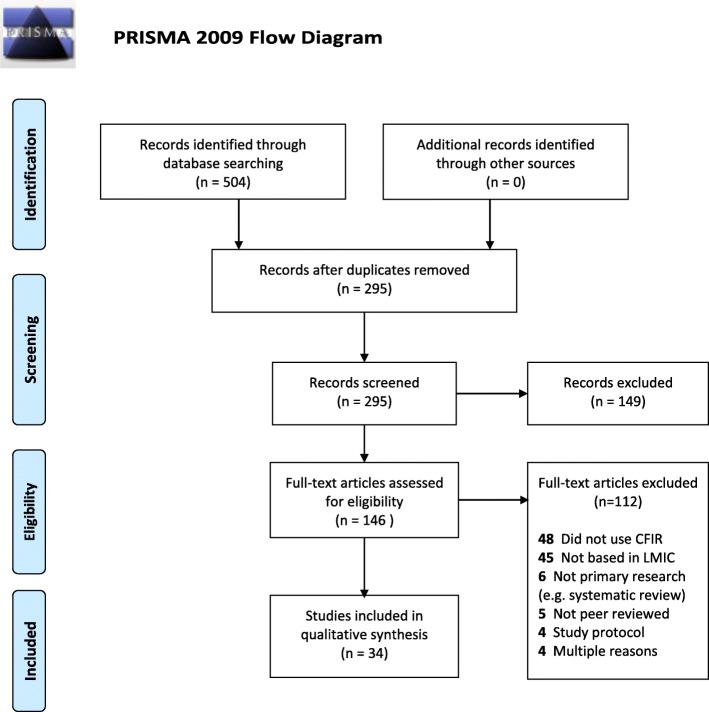
PRISMA flowchart of systematic review

Our database search yielded 504 articles. Of those, 209 were duplicate articles and were removed, leaving 295 unique articles. The titles and abstracts of these articles were reviewed, and 149 articles were excluded because they did not take place in an LMIC or were a protocol or editorial. We conducted a full-text review of the 146 remaining articles, of which 112 full-text articles were excluded: 48 were removed due to citing but not utilizing the CFIR, 45 were not based in an LMIC, five were not peer reviewed, six were not primary research (e.g., systematic review), four were a study protocol, and four met several exclusion criteria (Fig. 1).

The final sample included 34 studies (Table 1). The studies were written by 31 different first authors. Publication dates ranged from 2011 to 2019. The articles addressed implementation questions in 25 LMICs and territories, including South Africa (*n* = 6), Kenya (*n* = 5), Mozambique (*n* = 5), Pakistan (*n* = 3), Tanzania (*n* = 3), Zambia (*n* =3), Bangladesh (*n* = 2), Cameroon (*n* = 2), China (*n* = 2), Morocco (*n* = 2), Rwanda (*n* = 2), Uganda (*n* = 2), Vietnam (*n* = 2), Benin (*n* = 1), Chad (*n* = 1), Chile (*n* = 1), Côte d’Ivoire (*n* = 1), Ghana (*n* = 1), India (*n* = 1), Malawi (*n* = 1), Mexico (*n* = 1), Nepal (*n* = 1), Nigeria (*n* = 1), Thailand (*n* = 1), and the US Associated Pacific Islands (*n* = 1). One study conducted research in Canada simultaneously with research in Kenya, and one study did not specify a country of focus [11], as it described analytical findings associated with a global surgery working group [37]. Seven (21%) of the included studies were conducted in more than one LMIC.

**Table 1:** Summary of included studies

Author	Country	Health topic	Research objective(s)	Methods	Unit of analysis	Phase of CFIR application	Nature of CFIR application
Barac [10]	Chile, India, Pakistan, Bangladesh, Thailand, Vietnam, South Africa, and Nigeria	Typhoid	To identify typhoid-relevant interventions implemented between 1990 and 2015 and explore contextual factors perceived to be associated with their implementation	Mixed-methods	Organizations involved in implementation; Health policy and health system leaders at national or subnational levels	Post-implementation	Used to guide data collection; used to guide data analysis
Bardosh [11]	Canada, Kenya	HIV (Kenya)	To evaluate how a two-way SMS communication system to increase patient adherence to medication and engagement in care was perceived, diffused, and adopted in ongoing project sites	Qualitative design	Health providers in facilities involved in implementation	Mid-implementation	Used to guide data collection
Chu [12]	China	Hepatitis C virus (HCV)	To explore social and structural factors affecting HCV treatment access at an HIV treatment facility and methadone maintenance treatment centers to inform strategies for expanding access	Qualitative design	Patients benefiting from intervention	Post-implementation	Used to guide data analysis; used to interpret/contextualize findings
Cole [13]	Mozambique	Maternal health	To explore the contextual factors that may have contributed to observed increases in institutional deliveries from 2009-2014 in Nampula province.	Mixed-methods	Health providers in facilities involved in implementation; Patients benefiting from intervention	Post-implementation	Used to guide data collection; used to guide data analysis
Cooke [14]	Tanzania	Opioid treatment and HIV	To understand the contextual factors that influence the effectiveness of integrated methadone and anti-retroviral therapy implementation	Qualitative design	Patients benefiting from intervention; Health providers in facilities involved in implementation	Post-implementation	Used to guide data analysis
Dansereau [15]	Chad, Cameroon	Immunizations	To retrospectively evaluate the implementation of Gavi’s health system strengthening support and identify drivers of and barriers to implementation	Mixed-methods	Organizations involved in implementation	Post-implementation	Used to interpret/contextualize findings
Dogar [16]	Nepal, Pakistan	Tuberculosis (TB)	To describe challenges and lessons learned of implementing tobacco cessation in routine TB care	Qualitative design	Patients benefiting from intervention; health providers in facilities involved in implementation	Post-implementation	Used to interpret/contextualize findings
English [17]	Kenya	Pediatric inpatient care	To explore why a facility-based intervention to introduce care based on best-practice guidelines varied in effect across place and time	Mixed-methods	Organizations involved in implementation	Post-implementation	Used to interpret/contextualize findings
English [18]	Kenya	Pediatric inpatient care	To develop a system-oriented intervention to improve services for children in district hospitals	Qualitative design	Organizations involved in implementation	Pre-implementation	Used to frame/design the intervention
Gimbel [19]	Mozambique, Kenya, Cote d'Ivoire	HIV	To define the core and adaptable components of a facility-based intervention to address implementation challenges in prevention of mother to child transmission (PMTCT), and identify contextual influences that explain implementation heterogeneity	Qualitative design	Organizations involved in implementation	Post-implementation	Used to guide data collection; used to guide data analysis; used to interpret/contextualize findings
Gimbel [20]	Mozambique, Rwanda, and Zambia	Primary health care	To describes and categorize data quality assessment and improvement activities of a multi-country initiative and identify core intervention components and implementation strategy adaptations to improve data quality	Mixed-methods	Organizations involved in implementation	Post-implementation	Used to guide data collection, used to guide data analysis, used to interpret/contextualize findings
Gutierrez-Alba [21]	Mexico	Clinical practice guidelines generally	To identify and prioritize barriers and facilitators facing the implementation of Clinical Practice Guidelines in hospitals.	Qualitative design	Organizations involved in implementation	Mid-implementation	Used to guide data collection; used to guide data analysis; used to interpret/contextualize findings
Hosey [22]	US Associated Pacific Islands	Chronic disease	To describe the implementation and evaluation of a non-communicable disease (NCD) pilot project to systematically strengthen NCD health care quality and outcomes across five health systems	Mixed-methods	Organizations involved in implementation	Mid-implementation	Used to guide data analysis; used to interpret/contextualize findings
Huang [23]	Uganda	Pediatric mental health	To assess the feasibility and effectiveness of implementing professional development programs for early childhood teachers and determine if children with teachers exposed to professional development programs have better mental health outcomes	Mixed-methods	Health providers in facilities involved in implementation	Mid-implementation	Used to guide data collection
Jones [24]	Zambia	HIV	To identify predictors of a voluntary male medical circumcision program’s success or failure to create an “early warning” system that enables remedial action during implementation	Mixed-methods	Health providers in facilities involved in implementation	Mid-implementation	Used to guide data collection; used to guide data analysis’ used to interpret/contextualize findings
Landis-Lewis [25]	Malawi	HIV	To identify and describe barriers to using electronic medical record data for individualized audit and feedback for healthcare providers in Malawi and to consider how to design technology to overcome these barriers	Qualitative design	Health providers in facilities involved in implementation	Mid-implementation	Used to guide data collection
Malham [26]	Morocco	Maternal health	To identify the factors hindering full implementation of a national action plan to strengthen the professional role of midwives in two regions and to identify recommendations that could increase the effectiveness of the action plan	Qualitative design	Health providers in facilities involved in implementation	Mid-implementation	Used to guide data analysis; used to interpret/contextualize findings
Malham [27]	Morocco	Maternal health	To assess the extent to which a national action plan to strengthen the professional role of midwives was delivered in two regions, and the barriers and facilitators influencing implementation	Qualitative design	Health providers in facilities involved in implementation	Mid-implementation	Used to guide data analysis; used to interpret/contextualize findings
McRobie [28]	Uganda	HIV	To assess implementation of national HIV policies regarding testing, treatment, and retention at health facilities serving two health and demographic surveillance sites	Mixed-methods	Health providers in facilities involved in implementation	Pre-implementation	Used to frame/design the intervention
Myburgh [29]	South Africa	HIV	To identify barriers and facilitators in the implementation of an antiretrovirals electronic register at facility, sub-district, and district levels	Mixed-methods	Health providers in facilities involved in implementation	Post-implementation	Used to interpret/contextualize findings
Naidoo [30]	South Africa	HIV	To explore barriers and facilitators to implementation of community-based HIV programs in order to produce actionable findings to improve them	Qualitative design	Patients benefiting from intervention; health providers in facilities and in communities involved in implementation	Post-implementation	Used to interpret/contextualize findings
Nathavitharana [31]	Bangladesh	TB	To present operational data and discuss the challenges of implementing FAST (Find cases Actively, Separate safely and Treat effectively) as a TB transmission control strategy in health facilities	Qualitative design	Health providers in facilities involved in implementation	Post-implementation	Used to guide data analysis
Naude [32]	South Africa, Cameroon	Evidence based health policy generally	To describe the different contexts in which health policies are formulated and identify the facilitators and barriers to incorporating research evidence	Qualitative design	Health policy and health system leaders at national or subnational levels	Post-implementation	Used to interpret/contextualize findings
Petersen Williams [33]	South Africa	Maternal health	To investigate health care providers’ perceptionsof the acceptability and feasibility of providing screening, brief intervention, and referral to treatment to address substance use among pregnant women attending antenatal care	Qualitative design	Health providers in facilities involved in implementation	Pre-implementation	Used to frame/design the intervention; used to guide data collection
Phulkerd [34]	Thailand	Obesity	To identify barriers and potential facilitators to implementing regulations to restrict unhealthy radio and television food advertising to children and policies to promote healthier products	Qualitative design	Organizations involved in implementation	Pre-implementation	Used to guide data collection
Rodriguez [35]	South Africa	HIV	To identify barriers and facilitators in the implementation, uptake, and sustainability of PMTCT protocols in a rural areas	Qualitative design	Health providers in facilities involved in implementation; health policy and health system leaders at national or subnational levels; patients benefiting from intervention	Post-implementation	Used to guide data analysis; Used to interpret/contextualize findings
Rwabukwisi [36]	Ghana, Mozambique, Rwanda, Tanzania, and Zambia	Primary healthcare	To retrospectively evaluate a multi-country consortium aiming to implement and evaluate district-level health system strengthening interventions	Qualitative design	Organizations involved in implementation	Post-implementation	Used to guide data analysis
Saluja [37]	N/A	Surgery	To discuss key factors influencing implementation of national surgical planning in LMICs	Qualitative design	Organizations involved in implementation	Post-implementation	Used to guide data analysis; used to interpret/contextualize findings
Sax [38]	Pakistan	Primary healthcare (healthcare accreditation)	To identify perceived factors influencing introduction and adaptation of international healthcare accreditation to improve healthcare quality	Qualitative design	Organizations involved in implementation	Post-implementation	Used to guide data analysis
Shi [39]	China	Evidence-based public health generally	To assess implementation of evidence based public health and identify barriers to evidence based public health in the public sector	Qualitative design	Health providers in facilities involved in implementation; health policy and health system leaders at national or subnational levels	Pre-implementation	Used to frame/design the intervention; used to guide data collection; used to guide data analysis
Soi [40]	Mozambique	Human papillomavirus (HPV) vaccination	To identify implementation barriers and facilitators affecting the scale-up of HPV vaccination in Mozambique	Qualitative design	Health providers and educators in facilities and schools involved in implementation; health and education policy and health system leaders at national or subnational levels	Post-implementation	Used to guide data collection; used to guide data analysis
VanDevanter [41]	Vietnam	Tobacco cessation	To identify potential barriers and facilitators to implementing system changes to increase adoption of tobacco use treatment guidelines	Qualitative design	Health providers in facilities involved in implementation	Pre-implementation	Used to guide data collection; used to guide data analysis
Warren [42]	Kenya	Maternal health	To describe the complex processes, strengths, and challenges of an intervention aiming to address mistreatment during childbirth and promote respectful maternity care	Qualitative design	Patients benefiting from intervention; health providers in facilities involved in implementation	Post-implementation	Used to guide data analysis
White [43]	Benin	Surgical safety	To measure the sustainability of surgical safety checklist use and to evaluate the acceptability, adoption, appropriateness, feasibility and fidelity of nationwide checklist implementation, including penetration of the checklist into operating room culture	Mixed-methods	Health providers in facilities involved in implementation	Mid-implementation	Used to interpret/contextualize findings

There were 18 different health topics of focus across the articles, including HIV (*n* = 8), maternal health (*n* = 5), primary healthcare (*n* = 3), pediatric inpatient care (*n* = 2), surgery (*n* = 2), tuberculosis (*n* = 2), chronic disease (*n* = 1), clinical practice guidelines (*n* = 1), general evidence-based health policies (*n* = 1), general evidence-based public health practice (*n* = 1), hepatitis C (*n* = 1), HPV vaccination (*n* = 1), immunizations (*n* = 1), integrated HIV and opioid treatment (*n* = 1), obesity (*n* = 1), pediatric mental health (*n* = 1), tobacco cessation (*n* = 1), and typhoid (*n* = 1).

Qualitative study designs were most common, and quantitative assessments were relatively rare. Twenty-three (68%) of the studies employed qualitative study designs, while 11 (32%) were mixed methods designs. Common qualitative methods utilized across the studies included focus group discussions and key informant interviews. Mixed methods studies used review of financial records [15], routine facility, or surveillance indicators [13, 17, 22, 28, 43], health worker questionnaires or other quantitative study process indicators [10, 20, 23, 28, 29], or validated surveys to calculate measures such as organizational readiness and provider burnout [24] in conjunction with qualitative research. CFIR constructs can be scored quantitatively and compared across cases according to strength and valence [44]. Quantitative scoring of constructs was employed in three studies [19, 24, 40]. Another study created a quantitative questionnaire to align with CFIR constructs, in which participants were asked to rate CFIR constructs on a 5-point Likert scale from “very unimportant” to “very important” for implementation success [10].

The unit of analysis for most of the articles was health providers in facilities or communities involved in implementation (*n* = 19), followed by organizations (e.g., health facilities, district health offices) involved in implementation (*n* = 12), patients benefiting from the intervention (*n* = 7), and policymakers and health system leaders at national or subnational levels (*n* = 5). Nine of the studies focused upon more than one unit of analysis [10, 13, 14, 16, 30, 35, 39, 40, 42].

The CFIR can address different research questions depending upon the stage of implementation in which it is used. For example, pre-implementation, Shi et al. applied the CFIR to guide data collection and identify potential barriers to evidence-based public health in the public sector [39]. Mid-implementation, Malham et al. used the CFIR to assess the extent to which a national action plan to strengthen the professional role of midwives was delivered as well as the barriers and facilitators influencing implementation [27]. And, post-implementation, Rwabukwisi et al. applied the CFIR to retrospectively evaluate a multi-country consortium of district-level health system strengthening interventions [36]. Over half of the articles in this review applied the CFIR post-implementation (*n* = 20), 26% during mid-implementation (*n* = 9) and 18% pre-implementation (*n* = 6). Sixteen of the articles applied the CFIR for more than one research purpose, most of which were to guide data analysis (*n* = 19) or contextualize study findings (*n* = 16). Other CFIR applications include guiding data collection (*n* = 14) and framing or designing the intervention (*n* = 4). Damschroder et al. suggest that when the CFIR is applied post-implementation, it should be used to link determinants of implementation to targeted outcomes (e.g., intervention acceptability or effectiveness) [2]. However, only 6 (18%) studies reporting linking outcomes to specific CFIR constructs, all of which took place mid- or post-implementation [10, 17, 19, 21, 24, 40]. These papers identified relationships between specific CFIR constructs and measures of high and low fidelity to the intervention [19], measures of high and low uptake of the intervention [21, 24, 40], successful implementation generally [10], as well as variations in implementation success, including successes in intervention management, supervision, and facilitation [17].

**Fig. 2:**
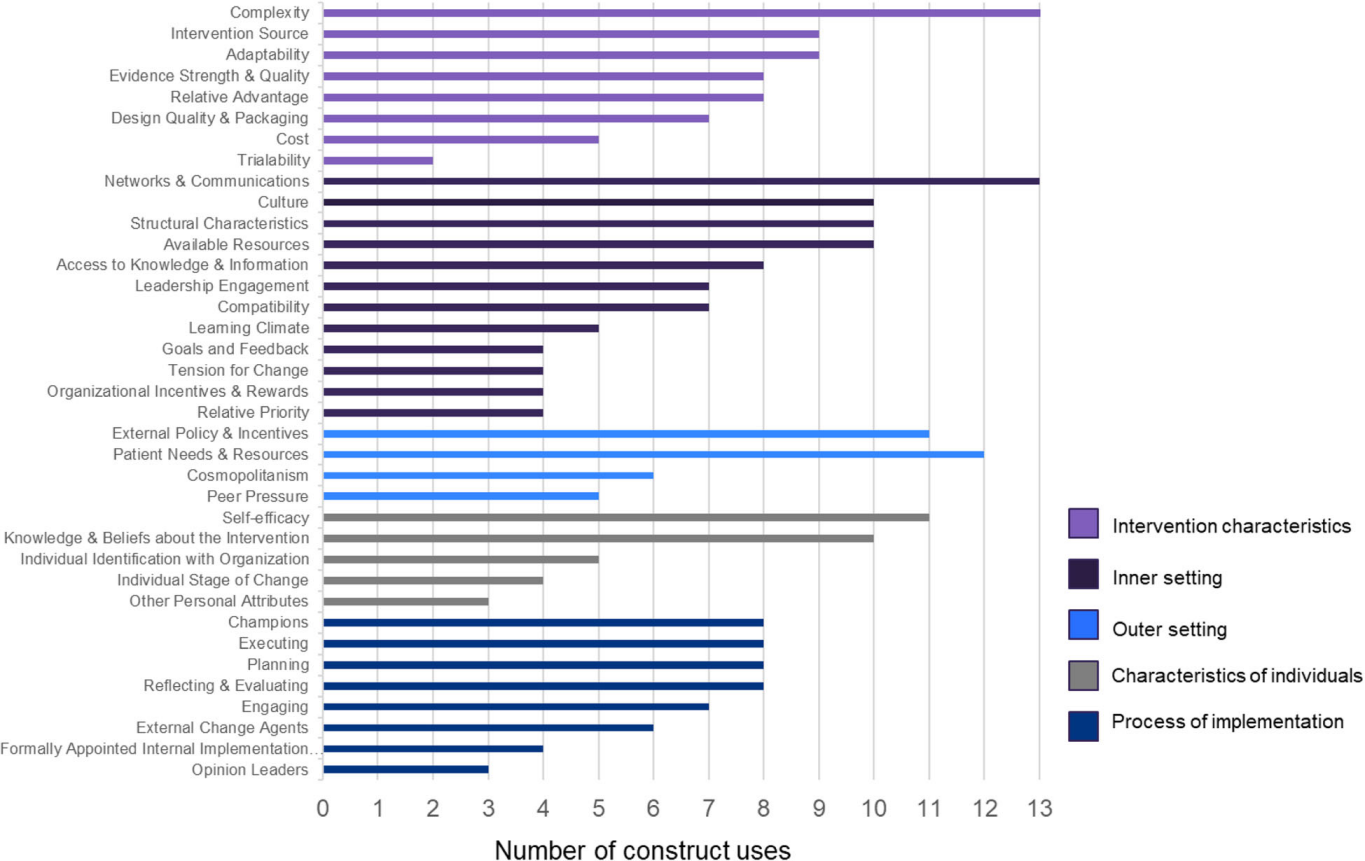
Count of CFIR constructs used in included systematic review studies, among studies reporting all constructs under consideration

Discussion about the use of the CFIR constructs varied widely across the studies. Eight studies (24%) only reported the domains used without their corresponding constructs. Four studies (12%) reported neither the domains nor the constructs used, and 17 (50%) studies utilized at least one construct from all five domains. One study reported constructs linked to study outcomes, but did not specify which constructs were initially considered within the analysis. *Complexity* and *networks and communication* were the mostly commonly used constructs, while *trialability* was the least commonly used construct (Fig. 2). Two (6%) of the studies reported examining all CFIR constructs [17, 24]. Two studies utilized constructs added to the CFIR within the process domain: *key stakeholders* and *innovation participants*. However, given that these constructs are not widely acknowledged as part of the CFIR, they are not included in the analysis for purposes of consistency [19, 40]. Damschroder et al. suggest that CFIR constructs be selected for use based on salience, level of application (ex., individual or health facility) and time point of application, and that researchers provide a rationale for why certain constructs were considered pertinent to the research question. Only 7 (21%) of the studies provided some justification for selecting the CFIR constructs used.

### Author survey

**Fig. 3:**
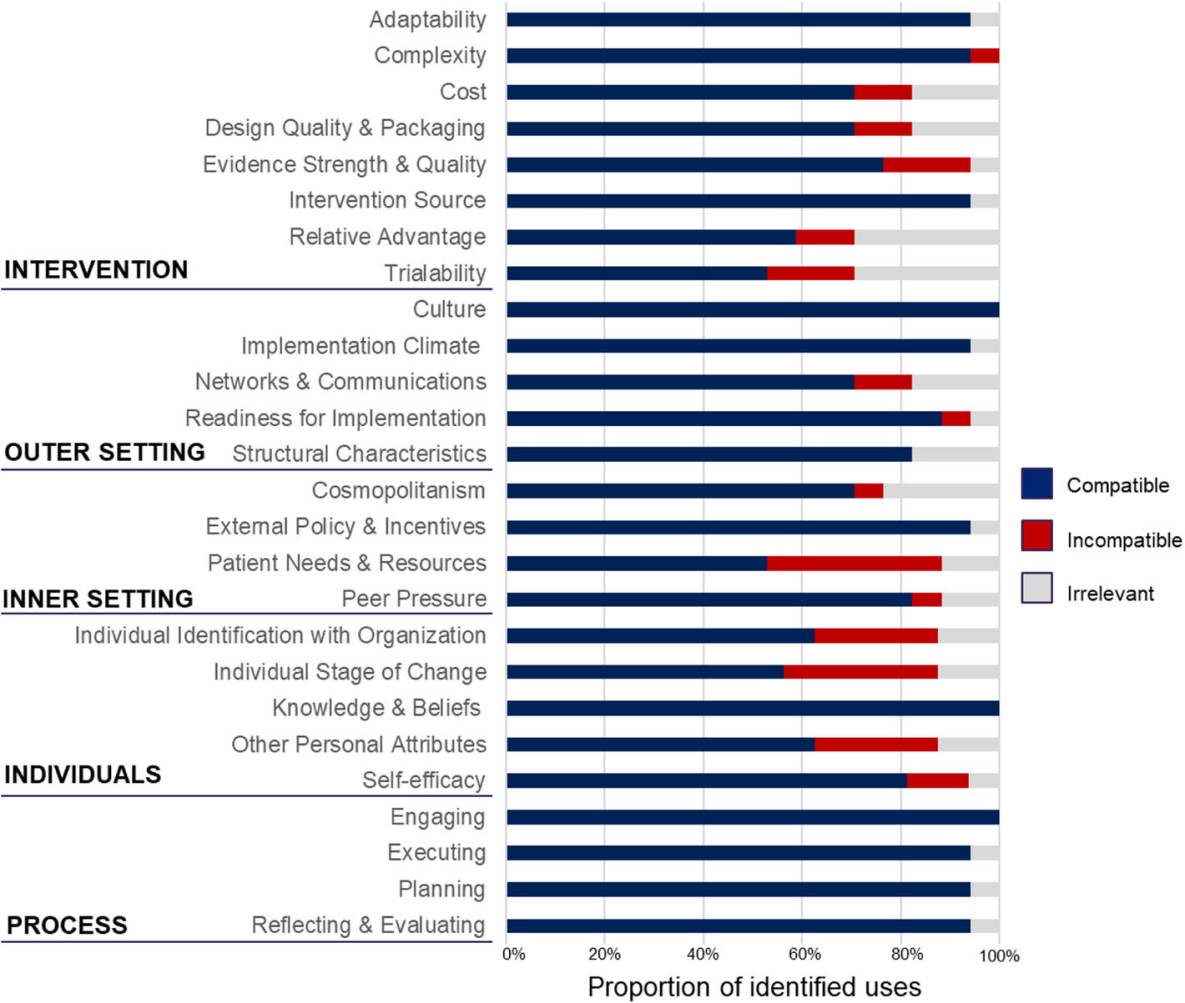
Responses from survey participants regarding compatibility of CFIR constructs

Nineteen (59%) of the 32 contacted authors participated in the survey. Most constructs were deemed by the authors to be compatible with use in LMICs (Fig. 3). Participating authors unanimously identified two constructs, organizational *culture* (inner setting domain), and *engaging* (process domain), as compatible with use in global implementation research. Some constructs were identified as irrelevant, largely due to the nature of the research question being asked. Only two constructs, *relative advantage* and *trialability*, both of which are within the intervention characteristics domain, were identified as irrelevant for use by five or more participating authors. Only two constructs, *patient needs and resources* (outer setting domain) and *individual stages of change* (characteristics of individual domain), were identified as incompatible with use by five or more participating authors.

Authors were requested to provide qualitative feedback regarding why specific constructs were considered incompatible with use. Regarding the construct of *patient needs and resources*, author responses followed two general themes. First, many studies employed interventions that took place at system levels broader than the facility (ex., district or national levels). Authors of these studies report that individual patient needs are not a compatible measure for health systems interventions inherently targeting systems-level barriers to care. Second, several authors reported that decision-making in the health systems in which their studies took place is not patient centered, and thus the construct is difficult to apply. Several authors added that organizational cultural or language barriers regarding practice norms made this construct particularly difficult to apply in an LMIC setting.

Authors who identified the construct of *individual stages of change* as incompatible cited that the construct is difficult to apply in health systems where the concept of individuality within a health care team is not compatible with the organizational culture. Many LMIC health care systems are more hierarchical than those in HICs, and the individual readiness of the health provider is less relevant. Tensions around individuality versus collectivism also influenced author perceptions of other CFIR constructs in the characteristics of the individual domain, such as *self-efficacy and individual identification with organization.*

Authors participating in the standardized questionnaire were asked to identify (1) circumstances in which the CFIR is not relevant for use in implementation research in LMICs, (2) possible adaptations or improvements that could be made to the CFIR for global implementation research, and (3) domains or constructs that should be added to improve relevancy. Responses to all three questions converged around prevailing themes of sub-organizational group or team-level influences on intervention delivery, as well as systems characteristics including perceived sustainability and scalability of interventions within the system. When asked about circumstances in which the CFIR may not be relevant to implementation science in LMICs, authors responses included the following:

In some settings health policy decisions are made from top down, and recipient will not have much option nor alternatives. In such conditions, CFIR individual and process domains might reflect skewed and over optimistic results – Author #29

Contexts vary largely such as the health systems and not only internally but the social norms, culture of the people and the political environment/economy...Therefore, it might be good to consider the macro-level factors as well – Author #32

When authors were asked about possible adaptations or improvements that could be made to the CFIR, most responses reinforced messaging regarding capturing health system dynamics influencing implementation. Authors had specific suggestions about constructs or domains that could be added to the CFIR to increase relevancy in LMICs, such as adding constructs that captured the resource constraints so often present in LMICs, as well as team-centered constructs that could focus on collective efficacy. Several authors also noted the need to include a systems-based domain, which could explore concepts of sustainability and long-term penetration of implementation activities within multiple levels of the health system. Author responses included the following:

It will be good if the CFIR can communicate more on how it can be used or applied in larger scale of actions such as implementation of national policy and strategy, not only at an intervention level – Author #22

More systems-based domains and constructs could be added in response to national and global actions such as accountability, governance & politics (both national and international) and legal and regulatory process. These factors play an important role in influencing the implementation of national policy – Author #23

It would be excellent if it could be adapted for use in researching health systems. In addition, if rather than, individuals there could be a domain for teams…I believe adding the domain of collective efficacy to characteristics of individuals would be useful – Author #38

### Modifications to CFIR for LMIC settings

**Fig. 4:**
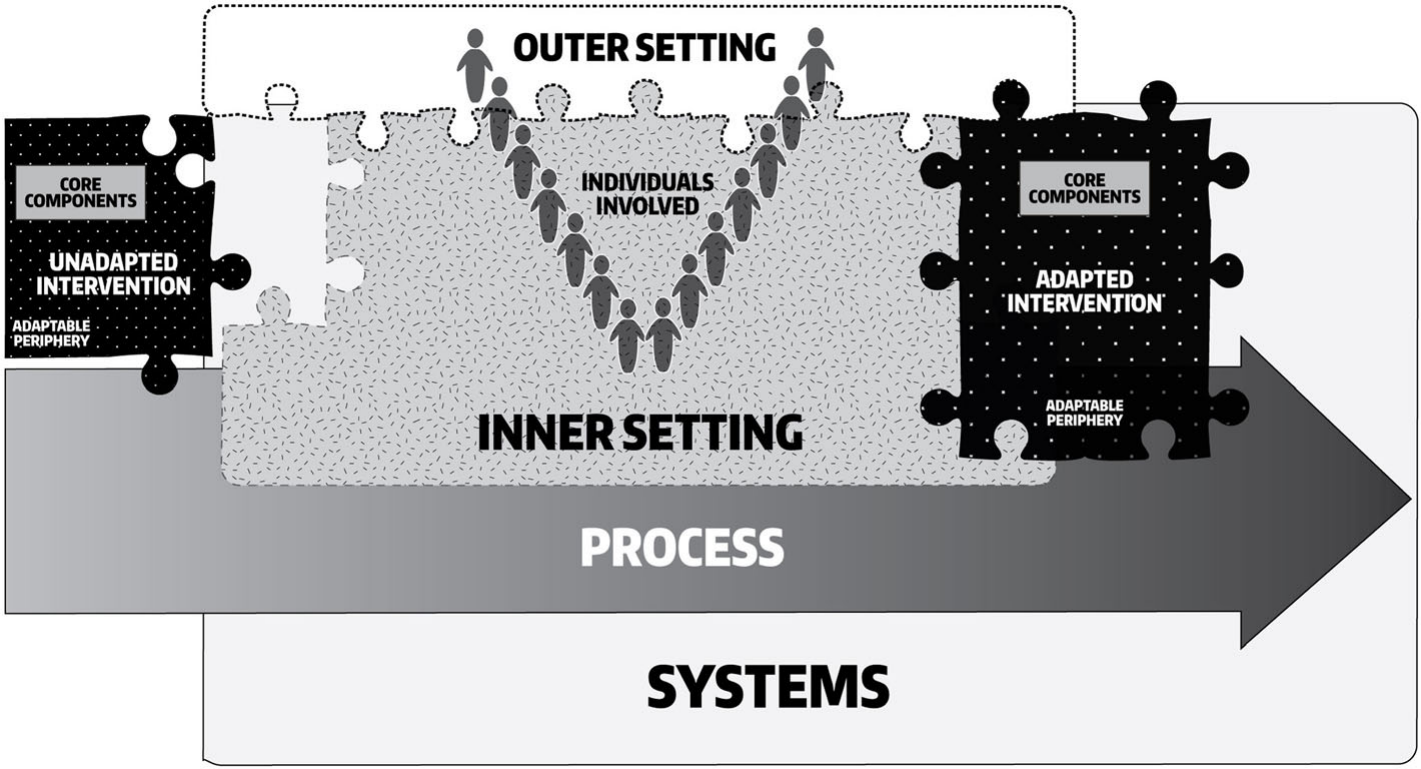
CFIR with new Characteristics of Systems domain

In order to address these perceived gaps in the CFIR taxonomy, as well as the experiences of review authors, we propose an additional domain called “Characteristics of Systems” to be added to the CFIR to increase its compatibility for use in LMICs. This domain includes constructs for, and related to, the relationship between key systems characteristics and implementation. Because proposed systems constructs have relational properties, they will inherently interact with existing constructs across domains. For example, the relative advantage of implementing an intervention may differ based on the perceived continuity of resources supporting implementation. Alternatively, the perceived sustainability of an intervention may be influenced by the adaptability of the intervention and the degree to which it can be tailored to meet local needs iteratively over time. As depicted in Fig. 4, a modified figure depicting the relationship of this additional domain with existing domains, each organization within a health system may have its own inner and outer setting, yet the system characteristics may be more ubiquitous across them. The Characteristics of Systems domain influences both the outer and inner settings in terms of how organizational culture and policy develop and, likewise, the two setting domains cyclically influence how health systems evolve or devolve over time through the actions and interactions of organizations within the system. The Characteristics of Systems domain has a similar relationship with the Individuals Involved and the Process of Implementation domains, wherein implementation determinants at the health systems level influence how individuals can or cannot engage in the process of intervention delivery. Likewise, the experiences of these individuals and implementation processes can engender health systems reforms that result in new implementation or innovation prospects.

We propose that the Characteristics of Systems domain contains six new constructs including: *external funding agent priorities, system architecture, resource source, resource continuity,* and *strategic policy alignment*. We also propose that several additional constructs be added to existing domains. These constructs include *perceived scalability* and *perceived sustainability* in the Characteristics of the Intervention domain, *team characteristics* and *collective efficacy* within the Inner Setting domain to account for the hierarchical practice norms more commonly present in LMICs, and *community characteristics* in the Outer Setting domain. Additionally, we suggest that *decision-making* be added to the Process domain. New constructs are defined in Table 2 and discussed below. Surveyed authors also proposed new constructs related to perceived feasibility and workload capacity; however, it was determined that perceived feasibility is well captured by existing CFIR constructs (ex., *complexity*, *adaptability*, and *cost*) while workload capacity is well captured by the existing Inner Setting construct, *compatibility*.

**Table 2:** Proposed additional constructs

Domain	Construct	Definition
Characteristics of Systems	Systems architecture	The administrative design of a health system or interacting systems that contribute to the health of the public (e.g., Ministries of Health, Education, Welfare, Sanitation, etc.) and that influence how programs are designed and/or implemented. This includes the nature of interactions across specific administrative level(s) that influence implementation. Examples of architectural attributes that may influence implementation include (de)centralized healthcare systems, remuneration and employment structures, governance, and supervisory structures, the role of health information systems, official roles and responsibilities of formal and informal health worker cadres.
	External funding agent priorities	Stakeholders’ perception regarding the degree to which funding agent preferences and priorities influence implementation. Examples may include mismatched priorities between donors and implementers, donor resources influencing implementer policy, or implementer policies influencing donor activities.
	Strategic policy alignment	The degree to which the perceived priorities and needs of relevant stakeholders are aligned with system policies and vice versa. Examples may include the perceived degree to which key stakeholders have input into strategic plans or that performance indicators accurately reflect health worker views of their professional responsibilities.
	Resource continuity	The presence of sufficient resources (financial, human, or material) over durations of time necessary for ongoing implementation at scale and without interruption or delays.
	Resource source	The origin of available resources used to test, launch, and sustain implementation. Plausible resource origins include domestic government resources directed to routine healthcare services, pilot programs or research, bilateral developmental aid, foreign governmental support for research, private foundation support, and multilateral organizations.
Characteristics of the Intervention	Perceived scalability	The perceived potential of implementation expansion so that the innovation/intervention is available across wider geographic or practice settings.
	Perceived sustainability	The perceived likelihood of continued use of program components and activities for the continued achievement of desirable program and population outcomes [45].
Inner Setting	Team characteristics	Features of a team including team composition, processes, and psycho-social traits. Examples of these features might include team diversity, interdependence/collaboration, and practice norms [46].
	Collective efficacy	A team’s shared belief in their capability to execute activities and achieve their common implementation goals.
Outer Setting	Community characteristics	The extent to which community characteristics affect the willingness or ability for organizations to engage in implementation. Community characteristics that might influence implementation include socio-cultural and religious features of healthcare consumers or health knowledge, attitudes, and beliefs influencing demand for healthcare services.
Process of Implementation	Decision-making	The type, duration and timing of the activities involved in making decisions about the intervention. Examples of decision-making characteristics that influence implementation may include decisions requiring highly bureaucratic approval systems, decisions that must be made far in advance or in conjunction with implementation, or even the absence of decision-making authority.

## Discussion

Our review identified 34 studies that utilized the CFIR in LMICs to address a variety of health topics ranging from specific diseases to introduction of evidence-based policies generally. This suggests rapid growth in the use of CFIR to support LMIC-based implementation research. Like the 2016 Kirk et al. review of CFIR articles predominantly from HIC settings, the studies in this review primarily utilized qualitative methods; however, several applied the CFIR with quantitative data as well, often to organize and condense programmatic monitoring in order to identify and understand implementation barriers or facilitators. Unlike the preceding review in which the unit of analysis was mainly the organization in which implementation occurred or the providers involved in implementation, studies included in this review most frequently took place at levels above the health facility, including district-level interventions, national-level policies, or even global advocacy efforts [3]. This reflects differences in the organization of healthcare delivery in many LMICs as compared to HICs; in these settings, services are frequently offered within government funded health facilities that are part of nationwide systems.

This review also found that the CFIR was applied at multiple stages of implementation. Before implementation begins, the CFIR can be used to investigate implementation barriers or facilitators prospectively, thereby informing program design as well as generating testable hypotheses that focus on specific constructs and their interrelationships. During implementation, the CFIR can be used to monitor implementation progress. And at the end of a study, the framework can be used to help explain success or failure in a post-implementation interpretive evaluation or determine degree of success in a summative evaluation. Constructs that may be most influential in the effective implementation of a specific intervention can be identified and linked to implementation or innovation outcomes of interest. During this phase, data can be analyzed using CFIR-guided codebooks and standard qualitative analysis methodologies, as well as through cross-case comparisons that facilitate rating of constructs to reflect the magnitude and valence of key CFIR constructs in influencing effective implementation [44]. In this review, we found that most studies applied the CFIR post-implementation in order to interpret and contextualize study findings. Very few studies, however, linked specific CFIR constructs to targeted study outcomes through purposeful data analysis or cross-case comparisons. A similar trend was observed in the Kirk et al. review where over half of studies in HICs applied the CFIR post-implementation. There continues to be room for more meaningful applications of the CFIR in guiding study design, monitoring implementation processes, data analysis, and outcome interpretation [3].

Over one-third of studies did not explicitly state what CFIR constructs were utilized. Of those that did report upon constructs, the two most commonly utilized constructs included *complexity* (Intervention Characteristics domain) and *networks and communication* (Inner Setting domain) while the least frequently utilized construct was *trialability* (Intervention Characteristics domain). However, it is important to note that construct usage frequency does not necessarily reflect constructs of highest utility, but rather constructs of greatest relevance to the research question at hand. Compared to the Kirk et al. review, the construct *intervention source* was used more frequently within LMICs (9 applications, 3% of total constructs used) as compared to within HICs (4 applications, 2% of total constructs used). Likewise *trialability* was used much less frequently in LMICs (2 applications, 0.7% of total constructs used) as opposed to in HICs (6 applications, 3% of total constructs used) [3]. These divergent patterns in construct use may reflect contextual differences between LMICs and HICs, and highlight the importance of evaluating and adapting implementation frameworks for use in LMIC settings.

Authors responding to standardized questionnaires reported that existing CFIR constructs are largely compatible for use in LMICs. Some constructs were identified as irrelevant or incompatible (*patient needs and resources* and *individual stages of change*) primarily for two reasons. Many constructs were simply not relevant to the specific research question at hand, while others were deemed incompatible with the organizational culture or structure of the health system. These responses reinforce efforts to adapt the CFIR to ensure that it is fit for purpose across settings. However, these responses also suggest an opportunity to review CFIR definitions to ensure that they are easily interpretable for individuals from a variety of disciplines. For example, although respondents stated that *patient needs and resources* was not a relevant construct for interventions targeting district or national levels, implementation at higher levels of the health system can still be patient-centered when patient needs and barriers, and facilitators to meet those needs are prioritized. The CFIR can be used to determine if failure to prioritize patient needs and resources, at any level and due to any reason, including socio-cultural norms, may influence implementation effectiveness.

Adapting the CFIR for specific uses or settings is not unprecedented. A 2014 report prepared for the Agency for Healthcare Research and Quality (AHRQ) by RTI International adapted the CFIR for use in three complex systems interventions: process redesign for improved efficiency and reduced costs, patient-centered medical homes, and care transitions between hospital and ambulatory care settings [47–49]. Across the three adapted frameworks, the report proposed the addition of two domains: a Measures of Implementation domain and an Outcomes domain to capture the effectiveness of implementation. Additionally, this effort renamed and redefined existing domains and constructs and proposed several dozen constructs specific to the systems interventions of focus. Proposed constructs included *radicalness* (Intervention Characteristics domain), *technological environment* (Outer Setting domain), *patient self-management infrastructure* (Inner Setting domain), *collective efficacy* (Characteristics of Individuals/Teams domain), *measurement capability and data availability* (Process domain), *reach* (Measures of Implementation domain), and *equitable* (Outcomes domain). While our review did not indicate a need to incorporate implementation measures and outcomes within the framework itself, it did corroborate the report’s description of key missing framework elements, namely sustainability and a focus on teams in addition to individuals.

Within a health system, each organization (e.g., a health facility) has its own distinct inner setting and outer setting. For proximal organizations (e.g., health facilities operating within the same district), the outer settings may be similar to one another or, in fact, the same. This phenomenon is best demonstrated in LMICs where governance is decentralized to local distinct subregions (i.e,. states or counties). In such countries, organizational outer settings across wider geographies or at different levels of the health system may exhibit significant variation due to differing health policies or practice norms in each subregion. For research in which the unit of analysis is above the organization, it may be necessary to consider other meta-characteristics of the health system. We propose the addition of a *Characteristics of Systems* domain to the current CFIR, which may help to resolve ambiguity regarding implementation determinants outside of the organization as well as distinguish between inner and outer settings within a hierarchical health system context. The proposed domain is relational, influencing and influenced by outer and inner settings, the process of implementation, the type and willingness of people to participate, and the progression through which an intervention is necessarily adapted. The proposed domain includes five constructs, several of which build upon existing models and theories, including the following: *systems architecture, external funding agent priorities, strategic policy alignment, resource continuity,* and *resource source*. We have also suggested that several constructs be added to existing domains, with the intention that additions should be made parsimoniously. These constructs include *perceived scalability* (Characteristics of the Intervention), *team characteristics* (Inner Setting)*, collective efficacy* (Inner Setting), *community characteristics* (Outer Setting), and *decision-making* (Process, sub-construct of *Executing*). These eleven constructs are summarized in Table 2 and the justification for adding these constructs is described in more detail in Appendix 2. The addition of these constructs is intended to augment the utility and comprehensiveness of the CFIR in LMICs.

## Conclusion

The purpose of this paper was to review existing applications of the CFIR in LMICs and learn from the reflections and experiences of authors who have utilized the CFIR in these settings. Limitations to this study include risk of incomplete retrieval of data from studies, or misinterpretation of reported study methodology. Our findings confirm that the CFIR is a popular and highly useful framework for global implementation science practitioners as it allows identification of implementation facilitators and barriers across settings. Constructs identified as more or less useful by authors often align with unique attributes of LMICs compared with HICs, such as more hierarchical versus more individualistic societies. To address the feedback provided, we have identified opportunities to adapt the CFIR for use in LMICs. Rather than redefine existing constructs, we have elected to maintain existing CFIR construct definitions so that past and future CFIR-based research operate within a standardized taxonomy. A newly proposed Characteristic of the System domain and constructs would provide global implementation science practitioners opportunities to account for health systems-level facilitators and barriers independent of the implementing organization. Newly proposed constructs have not yet been tested to ensure reliability and validity, which should be the focus of future measure development efforts.

### Supplementary information

**Additional file 1.** PRISMA Checklist.

## Data Availability

Abstracted data collected and analyzed during this study and described in this systematic review will be available from the corresponding author upon reasonable request. Qualitative responses from contacted authors are not available, as some of the information provided may be considered identifiable.

## Appendix 1

**Table 3:** CFIR domains and constructs [2]

**Domain 1: Characteristics of the Intervention**	
• Intervention source: Perception about whether intervention is externally or internally developed • Evidence Strength and Quality: Perception of the quality and validity of evidence supporting the belief that the intervention will have desired outcomes • Relative Advantage: Perception of the advantage of implementing the intervention versus an alternative solution • Adaptability: Degree to which an intervention can be tailored to meet the needs of an organization • Trialability: Ability to test the intervention on a small scale, and to reverse course if warranted • Complexity: Perceived difficulty of implementation • Design Quality and Packaging: Perceived excellence in how the intervention is bundled and presented • Cost: Cost of the intervention and costs associated with implementing the intervention	
**Domain 2: Outer Setting**	
• Patient Needs and Resources: Extent to which patient needs are accurately known and prioritized by the organization • Cosmopolitanism: Level of connectedness and networks with other organizations • Peer Pressure: Competitive pressure to implement an intervention • External Policy and Incentives: external strategies to spread interventions, including policy and regulations, mandates, recommendations and guidelines, etc.	
**Domain 3: Inner Setting**	
• Structural characteristics: Age, maturity, or size of the organization • Networks and Communication: Nature and quality of webs of social networks and the nature and quality of formal and informal communications within an organization • Culture: Norms, values, and basic assumptions of a given organization • Implementation climate: Relative priority of implementing the current intervention versus other competing priorities • Readiness for Implementation: Access to resources, knowledge, and information about the intervention	
**Domain 4: Individuals involved in implementation**	
• Knowledge and Beliefs about Intervention: Individual staff knowledge and attitude towards the intervention • Self-efficacy: An individual’s belief in their capabilities to execute the implementation • Individual State of Change: Phase an individual is in as he or she progresses toward skilled, enthusiastic, and sustained use of the intervention • Individual Identification with Organization: Individuals’ perception of the organization and their relationship and degree of commitment to the organization • Other Personal Attributes: Personal traits such as tolerance of ambiguity, intellectual ability, motivation, etc.	
**Domain 5: Process of implementation**	
• Planning: Planning for the implementation • Engaging: Engaging individuals in implementation processes • Executing: Executing the implementation plan • Reflecting and Evaluating: Reflecting and evaluating the progress of implementation	

## Appendix 2

### Description of newly proposed constructs

Implementation science foci include adoption or selection of evidence-based interventions, implementation of interventions using carefully selected implementation strategies, and scale-up of the interventions. Each focus, but particularly the third, requires consideration of unique, local health systems and national, regional, and global policies that influence broad delivery of evidence-based practices. However, as currently designed, the CFIR process domain ends at *reflecting and evaluating,* without conceptual next steps for expanding implementation. Author questionnaire respondents, in addition to CFIR utilizers from HICs, identified spread and scale as key missing constructs [50]. Thus, two newly proposed constructs include *perceived sustainability* (Characteristics of the Intervention) and *perceived scalability* (Characteristics of the Intervention)*.* The construct of *perceived sustainability* is intended to capture the perceived likelihood of continued use of program components and activities for the continued achievement of desirable program and population outcomes [45]. Sustainability is a key implementation outcome, and definitions of sustainability vary in the implementation literature [51, 52]. This construct, as presented, is intended to capture perceptions of sustainability at a health systems level throughout and after implementation, and not sustained behavior at an individual level. For studies focused solely on studying determinants or outcomes of sustainability, we suggest that researchers utilize models and frameworks focused explicitly on exploring the dynamics of sustainability [53, 54].

The CFIR can currently be applied to anticipate implementation needs through exploration of constructs such as *planning, executing, and reflecting and evaluating.* However, the newly proposed construct *scalability* is intended to capture the perceived potential of implementation expansion so that that the innovation/intervention is available across wider geographic or practice settings. This construct would be appropriate for use to capture perceptions regarding why an intervention could or could not scale, and how implementation could be modified or optimized to enhance replicability of core intervention components. The construct reflects issues and opportunities related to health system infrastructure and capacity, and are thus inherently linked to constructs within all CFIR domains. However, if researchers aim to study scale-up as an outcome, as opposed to a determinant, more comprehensive scalability frameworks are recommended [55]. It is our hope that by adding constructs of both *perceived sustainability* and *perceived scalability* to the CFIR, these important features of implementation will be more frequently and more fully explored as determinants of implementation by global implementation researchers.

Several other constructs were proposed within the new Characteristics of Systems domain. The *systems architecture* construct addresses how the many organizations operating within a health system, each with their own inner and outer settings, interact with one another to influence implementation. The *systems architecture* construct thus inherently builds upon *structural characteristics* in the Inner Setting. This construct may be particularly useful for multi-level interventions targeting different implementation mechanisms and administrative levels of a health system and could be used to stratify and apply constructs across levels. *Systems architecture* may also be particularly useful when conducting cross-country analyses, as the design of a health system, including the degree of decentralization, often influences which implementation strategies are most effective in a given setting. Two resource-specific constructs are also included within the domain, including *resource source* and *resource continuity*, both of which reflect unique determinants of implementation outcomes in LMICs. Together, they highlight the flow of resources outside of the organization, and the economic and strategic trends that influence where and how these resources are made available to the organization. They also complement *available resources,* a construct found in the Inner Setting domain, which describes the level of resources available to an organization for implementation.

Linking the Characteristics of Systems, Outer Setting, and Intervention domains is the construct of *external funding agent priorities*, which highlights how funding agents influence both intervention design and the implementation environment. This construct complements but is distinct from *external policies and incentives* within the Outer Setting domain as *external funding agent priorities* can often influence how interventions are derived, designed, prioritized and delivered, thus permeating multiple existing CFIR domains. The construct of *strategic policy alignment* was added in response to requests for constructs that capture how the priorities of various stakeholders and organizations do or do not reflect local governmental health policies. This construct also expands upon *external policies and incentives* by identifying how a specific policy or guideline is introduced or delivered within the health system and can be used to highlight policies or guidelines that are in conflict with one another or do not align with long-term health planning documents.

In response to author feedback and influenced by the Integrated Team Effectiveness Model (ITEM), we also propose a construct for *team characteristics* in the Inner Setting domain to capture how interpersonal norms and dynamics influence implementation within an organization [46]. *Team characteristics* are distinct from the existing construct of *learning climate*, which is focused primarily on skill development and growth. Rather, *team characteristics* can capture and describe interpersonal norms that influence implementation within hierarchical health systems, including concepts such as team accountability, workflow, and allocation of responsibilities. *Team characteristics* are also related to the newly proposed construct of *collective efficacy,* a team’s shared belief in their capability to execute activities and achieve their common implementation goals.

This research also highlighted the need to account for *community characteristics* as a new construct in the Outer Setting domain because communities, community mobilization, and community infrastructure are the foundation of an effective, decentralized public health system. This construct recognizes the effect that communities can have on individual patients, health providers, and organizations by influencing patient care seeking as well as intervention design, delivery processes, and supportive policies. Notably, this construct would also apply in circumstances in which community stigma or resistance might influence design or uptake of an intervention.

Finally, the construct *decision-making* is proposed within the Process domain as a sub-construct of the existing construct, *executing.* While *executing* reflects implementation fidelity, timeliness, and engagement, *decision-making* distinctly emphasizes the many actions that must be taken in the process of making timely and effective decisions about implementation. *Decision-making* is highly linked to *reflecting and evaluating* within the Process domain, in that decisions will be made in light of or despite of implementation benchmarks, and likewise implementation monitoring is often designed to inform relevant decisions. Notably, this construct was also proposed within the AHRQ-RTI CFIR adaptation [47].
